# Postpartum Blood Loss in COVID-19 Patients—Propensity Score Matched Analysis

**DOI:** 10.3390/biomedicines10102517

**Published:** 2022-10-09

**Authors:** Marcin Januszewski, Małgorzata Santor-Zaczyńska, Laura Ziuzia-Januszewska, Michał Kudan, Alicja A. Jakimiuk, Waldemar Wierzba, Artur J. Jakimiuk

**Affiliations:** 1 Department of Obstetrics and Gynecology, Central Clinical Hospital of the Ministry of Interior and Administration, 02-507 Warsaw, Poland; 2 Department of Otolaryngology, Central Clinical Hospital of the Ministry of Interior and Administration, 02-507 Warsaw, Poland; 3 Department of Plastic Surgery, Central Clinical Hospital of the Ministry of Interior and Administration, 02-507 Warsaw, Poland; 4 Satellite Campus in Warsaw, University of Humanities and Economics in Lodz, 01-513 Warsaw, Poland; 5 Center for Reproductive Health, Institute of Mother and Child, 01-211 Warsaw, Poland

**Keywords:** SARS-CoV-2, postpartum hemorrhage, COVID-19, obstetric, labor, bleeding, pregnancy, coagulation

## Abstract

The aim of this study was to compare the estimated blood loss and the frequency of obstetric hemorrhage among pregnant women with and without COVID-19 infection. The study was carried out in the Department of Obstetrics and Gynecology, at the Central Clinical Hospital of the Ministry of the Interior and Administration in Warsaw, Poland. From 15 May 2020 to 26 April 2021, a total of 224 parturients with COVID-19 infection were admitted for labor. The control group consisted of 300 randomly recruited pre-pandemic deliveries that took place between 15 May 2019 and 26 April 2020 at the Department. The primary outcome was the presence of postpartum hemorrhage, defined as an estimated blood loss of ≥500 mL within 24 h after birth or the need to transfuse 2 or more units of packed red blood cells (pRBCs). Secondary outcomes were the difference between hemoglobin and hematocrit levels at 24 h postpartum, the number of pRBCs units transfused, and the need for transperitoneal drainage. After applying the propensity-score-matching procedure for postpartum bleeding risk factors, 325 eligible patients were included in the final analysis, divided into 203 COVID-19 positive and 122 COVID-19 negative prepandemic deliveries. SARS-CoV-2 infected patients were characterized by a longer activated partial thromboplastin time (APTT), a reduced prothrombin time (PT), and lower platelet count at initial presentation. COVID-19 deliveries were found to be associated with a higher frequency of postpartum hemorrhage, an increased estimated blood loss, the more frequent use of peritoneal drainage, and more pRBCs units transfused. During the pandemic, an increased risk of postpartum hemorrhage posed another threat to SARS-CoV-2 infected pregnant women. It is essential to be aware of this when approaching COVID-19 delivery and to implement efficient preventative methods.

## 1. Introduction

Postpartum hemorrhage (PPH) is a leading cause of maternal death in well and poorly developed countries, with mortality oscillating from 0,6 to 20% [1]. Its incidence is about 3%, with an upward trend, and is divided into primary, occurring within 24 h postpartum, and secondary, from 24 h to 12 weeks postpartum [2,3]. However, the definition of PPH is not well established and differs among various organizations [4,5,6]. Many risk factors and stratification systems have been outlined in the literature [7,8]. The ongoing COVID-19 pandemic creates an urgent need to understand the rate of postpartum bleeding in SARS-CoV-2 infected parturients and thus improve preventative efforts in this cohort, including blood products reservation and tranexamic acid administration [9,10]. During pregnancy, a prothrombotic state is observed, which is manifested by increased levels of fibrinogen and D-dimer with a lower platelet count. Moreover, plasma levels of coagulation factors are increased, resulting in reduced activated partial thromboplastin time (APTT) and prothrombin time (PT). In the general population, coagulation disorders in the course of SARS-CoV-2 infection are mainly observed in moderate, severe, or critical disease [11,12,13] and may manifest as both thrombotic and hemorrhagic complications. Indeed, in SARS-CoV-2 infection, microthrombosis in the lungs, heart, brain, and kidney, as well as venous thromboembolism (VTE), may occur. The concurrent activation of the coagulation and fibrinolysis cascades, further induced by sepsis, causes the consumption of coagulation factors and promotes hemorrhagic events. COVID-19-associated coagulopathy is therefore characterized by increased levels of fibrinogen and D-Dimer, normal or lower platelet count, prolonged APTT, and normal to prolonged PT. In a mild course of an illness, SARS-CoV-2 affects the platelet count, resulting in mild thrombocytopenia with increased platelet consumption [14] in correlation with subclinical placental infection [15], and it may play a role in increased postpartum blood loss. Therefore, the assessment of coagulation status in pregnant COVID-19 patients may be impaired, and laboratory results may be misinterpreted [11,12,16] in a correlation with a clinical outcome.

The aim of this study was to assess whether the estimated blood loss and the frequency of obstetric hemorrhage differed among pregnant women with and without COVID-19 infection.

## 2. Materials and Methods

### 2.1. The Study Population

This retrospective, single-centered comparative study was conducted in the Department of Obstetrics and Gynecology, at the Central Clinical Hospital of the Ministry of the Interior and Administration in Warsaw, Poland. From 15 May 2020 to 26 April 2021, a total of 224 pregnant women with COVID-19 infection were admitted for labor or induction of labor. The control group consisted of 300 randomly recruited pre-pandemic deliveries that took place between 15 May 2019 and 26 April 2020 at the clinic. The research was approved by the Bioethics Committee of the Central Clinical Hospital of the Ministry of the Interior and Administration in Warsaw (decision nr 104/2021). The primary outcome was the presence of PPH, defined as an estimated blood loss of ≥500 mL within 24 h after birth or the need to transfuse 2 or more units of packed red blood cells (pRBCs). Secondary outcomes were the difference measured in estimated blood loss, the difference between hemoglobin and hematocrit levels 24 h postpartum, the number of blood units transfused, and the need for transperitoneal drainage. Estimated blood loss was quantified by trained personnel (surgical drape + soaked material count for cesarian section and under-buttock calibrated drape + soaked material count for vaginal delivery).

### 2.2. Inclusion and Exclusion Criteria

Pregnant women were included if they were admitted for labor or the induction of labor. SARS-CoV-2 infection was confirmed either by a positive antigen test or by a positive real-time reverse transcriptase-polymerase chain reaction (rRT-PCR) assay performed no more than 13 days before the admission.

### 2.3. Study Procedures

We included patients with positive results of rRT-PCR or rapid antigen tests, characterized by a sensitivity of ≥80% and a specificity of ≥97%, thus meeting the conditions for use for diagnostic purposes, including the Panbio COVID-19 AG Rapid Test Device (Abbott, Abbot Rapid Diagnostics Jena GmbH, Jena, Germany) and Bioeasy 2019-nCoV Ag Fluorescence (Shenzhen Bioeasy Biotechnology Co. Ltd., Shenzhen, China). The following variables were analyzed: maternal age, body mass index (BMI), pre-existing medical conditions (diabetes mellitus, preeclampsia, hypothyroidism), history of PPH, pregnancy status, and gestational age at birth. Data regarding risk factors significantly associated with hemorrhage were collected, including the delivery route, retained placenta or membranes, adherent placenta, placental abruption, failure of labor progress, instrumental delivery, newborn weight, induction of labor, high parity (≥3), multiple gestation, chorioamnionitis, leiomyomas, uterus defects, previous uterine surgeries, history of known coagulation disorders, use of antithrombotic drugs (history of aspirin administration within 7 days and, in case of low-molecular-weight heparin (LMWH), 12 h for prophylactic and 24 h for treatment dose), prepartum thrombocyte count, and prepartum hematocrit level. At admission, all women underwent blood count, coagulation, and biochemical tests, and 24 h after delivery, blood count was performed.

### 2.4. Statistical Analysis

Data missing in no more than 5% were filled by the random-forest method, performed with the “missForest” package from the CRAN repository [17] in R software. Before the matching procedure, univariable and multivariable logistic regression analyses were performed to determine the association of the patient‘s characteristics and PPH. To perform the propensity score matching, the “MatchIt” package from the CRAN repository [18] in the R software was used. For the comparison of two groups, the Mann–Whitney U test was used for continuous, non-normally distributed variables and the Chi Square test was performed for categorical variables. When the expected number of frequencies in the Chi square test was low, Fisher’s exact test was applied. A level of *p* < 0.05 was used to determine statistical significance.

## 3. Results

There were no cases with a history of PPH, uterus defects, placenta praevia, and an adherent placenta.

Risk factors for PPH such as uterine myomas, twin pregnancy, placenta praevia, retained placenta or membranes, placental abruption, chorioamnionitis, uterus defects, and coagulation disorders were removed from the analysis due to their low incidence.

The patients’ characteristics before matching are presented in the Table S1. PPH was associated with COVID-19 in univariate (OR = 4.19, 95%CI 1.44–15.1, *p* = 0.014) and multivariate (OR = 5.92, 95%CI 1.82–23.9, *p* = 0.006) logistic regression analysis (Table S2).

We matched the groups according to the following variables: BMI, the presence of preeclampsia, the route of delivery, gestational age, multiparity (≥3), previous operations on the uterus, anticoagulant use, the induction of labor, failure to labor progress, hematocrit level, and fetal weight. After applying propensity score matching, 325 eligible patients were included in the final analysis, divided into 203 COVID-19-positive and 122 COVID-19-negative prepandemic deliveries. SARS-CoV-2-positive patients when compared to the matched control group of prepandemic patients had longer APTT (<0.001), reduced INR (*p* < 0.001), and lower platelet count (*p* < 0.011) at initial presentation.

In patients with COVID-19, deliveries were found to be associated with a higher frequency of PPH-1 (0.8%) vs. 11 (5.4%) *p* = 0.035, an increased estimated blood loss-median = 350 vs. 350, *p* = 0.021, more frequent use of peritoneal drainage 0 vs. 47 (23%) *p* < 0.001, and more pRBCs units transfused-median = 1 vs. 1 *p* = 0.009. However, we did not find a greater difference between pre- and postpartum hemoglobin and hematocrit levels in COVID-19 patients compared with the matched control group of non-COVID patients.

The patients’ characteristics after matching procedure are present in Table 1.

## 4. Discussion

COVID-19 labors were associated with an increased incidence of PPH (0.8% vs. 5.4% *p* = 0.035) in univariate (OR = 4.19, 95%CI 1.44–15.1, *p* = 0.014) and multivariate (OR = 5.92, 95%CI 1.82–23.9, *p* = 0.006) logistic regression analysis as well as more frequent need of peritoneal drainage usage (0% vs. 23% *p* < 0.001). Moreover, after propensity score matching COVID-19 deliveries were found to be characterized by increased estimated blood loss (*p* = 0.021) and more units of pRBCs transfused (*p* = 0.009), the difference between pre- and postpartum hemoglobin and hematocrit concentration in COVID-19 compared with matched control group of non-COVID deliveries emerged as a poor indicator of heavy blood loss.

Several studies examining blood loss associated with COVID-19 labors have had contradicting results. An early case report outlined a possible link between the COVID-19 infection and the coagulopathy resulting in PPH [19]. In a meta-analysis including 121 studies involving COVID-19 pregnant patients by Jafari et al., the authors revealed a high prevalence of PPH among COVID-19 pregnant patients, with an incidence of 54.5% [20]. That study showed a 10-fold greater incidence rate than our work and seemed to be compelling. However, no transparent definition of PPH was outlined in that work.

In another work made by Hcini et al. including 507 pregnant women, among which 137 were Sars-Cov-2 infected, the authors outlined a greater incidence of PPH defined as a blood loss of >500 mL (14.2% vs. 7.2%, RR 2.0 (95%CI 1.1–3.4) and a transfusion need 5.5% vs. 1.1%, RR 4.9 (1.5–16.6)) in COVID-19 patients [21].

Also in line with those findings have been the conclusions from a recent US national cohort study involving 78,283 pregnancies and 2655 documented SARS-CoV-2 infections [22]. Regan AK et al. indicated that COVID-19 during pregnancy had been associated with a two-fold higher risk of PPH (aHR 2.03, 95% CI 1.6–2.63) with incidence similar to ours—5.4%. In that work, the adjusted hazard models, including maternal age, race, annual income, presence of pre-existing medical condition (yes/no), and week of pregnancy only partially indicated the use of heavy postpartum bleeding risk factors [22].

Moreover, in another national retrospective cohort study from France, Epelboin et al. found a higher frequency of PPH in the COVID-19 group compared with the non-COVID-19 patients (10.0% versus 5.7%, *p<* 0.001) with the increased adjusted odds ratio (aOR = 1.7, 95% CI (1.4 to 2.1)). In that work, similar to our study, the criterium for PPH was applied [23] and the multivariable analysis was performed with the adjustment to maternal age, BMI, active smoking, parity, history of diabetes or hypertension, multiple pregnancy, and ART conception.

Furthermore, the most recent study concerning the morbidity from obstetric complications in COVID-19 cohort (2352 births) in comparison to non-infected controls (11,752 births) also revealed that SARS-CoV-2 infection had been significantly associated with PPH (2.6% vs. 2.4%), with an adjusted relative risk of 1.13 (0.83 to 1.53, 95% CI), including maternal age, body mass index, the presence of any comorbidity, and obstetric history as clinically relevant covariates [24]. In this study, PPH was categorized as a need of transfusion of four or more units of packed red blood cells or surgical/radiologic interventions.

One paper, however, assessed the risk of obstetric hemorrhage related to COVID-19 diagnosis, including 760 COVID- and 53 COVID + births, and indicated no increased risk of obstetric hemorrhage and peripartum quantitative blood loss (QBL) among SARS-CoV-2 infected pregnant women [20]. The authors of that study defined obstetric hemorrhage as any QBL > 1000 mL and revealed an incidence of 11.3% PPH among COVID positive vs. 18.4% COVID negative deliveries. Multivariable logistic regression revealed an odds ratio 0.41 (0.17–1.04) adjusted for delivery mode, BMI, and the presence of intraamniotic infection [25]. Similarly, earlier work made by Liao J et al. assessing the prevalence of PPH showed no difference between 10 SARS-CoV-2 infected and 53 non-infected vaginal deliveries [26]. Additionally, Zhang L et al. reported no significant differences in the intraoperative blood loss in a retrospective comparison of the pregnancy outcomes between 16 women with COVID-19 and 45 women without COVID-19 [27]. Those two works mentioned above were characterized by insufficient statistical power due to small group sample. Moreover, Son M et al. compared women classified as SARS-CoV-2- negative (100,635 births) with those who tested positive for SARS-CoV-2 infection (7432 births) and found no significant differences in the frequency of PPH (3.4% vs. 3.1%, standardized difference = 0.019) [28].

Nevertheless, none of the aforementioned studies, both supporting and contradicting our thesis, used a propensity score matching of the groups stratified by clinically relevant covariates. In times of pandemic, when supervising and taking care of pregnant women are challenging, the increased risk of PPH seems to be another threat to their health and life.

We believe that the appropriate preventive measures, including blood reserve, prolonged close postpartum surveillance, and additional uterotonics usage, should be implemented at parturients with COVID-19, particularly those with other risk factors for PPH. Moreover, a SARS-CoV-2 infection may be considered as an independent obstetric hemorrhage risk factor.

In COVID-19 patients without otherwise explained PPH, fresh frozen plasma, fibrinogen, or tranexamic acid should be considered due to their efficiency in a supplementing disrupted coagulation cascade. Tranexamic acid, however, should be avoided in DIC [12,29].

The limitations of our study include its single-center nature, homogenous group of patients, and imperfect measuring methods.

## 5. Conclusions

We observed that mothers with COVID-19 have an increased risk of PPH. The early identification of patients at risk of developing in-hospital complications is essential in terms of the effective use of health resources.

## Figures and Tables

**Table 1:** Patient characteristics after matching procedure.

Variable	Patients (N = 325)		*p*-Value
	COVID-19 Cases (N = 203)	Controls (N = 122)	
Labor ≥3	31 (15%)	20 (16%)	0.788 *
Previous operation on uterus	52 (26%)	41 (34%)	0.123 *
Anticoagulant use	11 (5.4%)	9 (7.4%)	0.477 *
Induction of labor	34 (17%)	26 (21%)	0.305 *
Fail to labor progress	14 (6.9%)	10 (8.2%)	0.664 *
Diabetes	31 (15%)	20 (16%)	0.788 *
Preeclampsia	18 (8.9%)	14 (11%)	0.445 *
Hypothyroidism	43 (21%)	26 (21%)	0.978 *
Cesarean section	134 (66%)	78 (64%)	0.704 *
Gestational age (weeks)	39 (38; 40)	39 (38; 40)	0.456 ***
BMI (kg/m2)	28.63 (26.08; 31.18)	27.74 (26.31; 31.09)	0.468 ***
Hematocrit count before birth (%)	37.80 (35.70; 39.75)	37.60 (35.92; 40.08)	0.932 ***
Duration of hospitalization (days)	6 (5; 6.5)	6 (5; 6.75)	0.935 ***
Blood units transfused (N)	1 (1; 1)	1 (1; 1)	0.009 ***
Peritoneal drainage	47 (23%)	0 (0%)	<0.001 **
Postpartum hemorrhage	11 (5.4%)	1 (0.8%)	0.035 **
Estimated blood loss (ml)	350 (300; 400)	350 (300; 400)	0.021 ***
Platelet count (10^3^/µl)	196 (156; 236.5)	210 (181; 241.25)	0.011 ***
Hemoglobin concentration before birth (g/dl)	13 (12.15; 13.6)	12.4 (11.9; 13.3)	0.003 ***
Hemoglobin concentration after birth (g/dl)	11.6 (10.75; 12.5)	11.15 (10.50, 11.90)	0.005 ***
Decrease in hemoglobin concentration (g/dl)	1.2 (1.8; 0.6)	1.25 (2; 0.52)	0.865 ***
Hematocrit concentration after birth (%)	34.4 (32.1; 36.85)	33.75 (31.7; 36.68)	0.341 ***
Hematocrit concentration loss (%)	3 (5; 1.3)	3.5 (5.62; 2)	0.037 ***
Leukocyte count (10^3^/µl)	9.20 (7.44; 10.85)	10.53 (9.29; 12.93)	<0.001 ***
Lymphocyte count (10^3^/µl)	1.59 (1.20; 1.93)	1.87 (1.49; 2.2)	<0.001 ***
Neutrophile count (10^3^/µl)	6.89 (5.24; 8.4)	7.61 (6.49; 9.68)	<0.001 ***
Sodium serum level (mmol/L)	139.00 (137; 140)	138.07 (137; 140)	0.993 ***
Potassium serum level (mmol/L)	4.09 (3.88; 4.28)	4.15 (3.98; 4.28)	0.122 ***
APTT (seconds)	29.90 (27.4; 32.4)	28 (26.50; 29.85)	<0.001 ***
INR	0.95 (0.92; 0.98)	0.97 (0.94; 1.02)	<0.001 ***
Birth weight (g)	3,450 (3,090; 3,770)	3,375 (3,097.5; 3,730)	0.916 ***

* Pearson’s Chi-squared test; ** Fisher’s exact test; *** Mann-Whitney U test. Categorical variables are presented as N (%) and continuous variables are presented as Median (IQR).

## Data Availability

The datasets generated and/or analyzed during the current study are not publicly available due to our policy but are available from the corresponding author upon reasonable request.

## Supplementary Materials

The following supporting information can be downloaded at: https://www.mdpi.com/article/10.3390/biomedicines10102517/s1, Table S1. Patient characteristics before matching procedure; Table S2. Univariate and multivariate logistic regression before propensity score matching.
