# Supplementary material for: Postpartum Blood Loss in COVID-19 Patients—Propensity Score Matched Analysis
Source: Biomedicines. 2022 Oct 9;10(10):2517. doi: 10.3390/biomedicines10102517 (PMC9599331; doi:10.3390/biomedicines10102517)
Supplement: Supplementary file 1 [file biomedicines-10-02517-s001.zip › biomedicines-1882222-supplementary.pdf]

Table S1. Patient characteristics before matching procedure.

Variable	Patients (N=325)		p-value
	COVID-19 Cases (N = 203)	Controls (N=122)	
Labor ≥3	31 (15%)	20 (16%)	0.788*
Previous operation on uterus	52 (26%)	41 (34%)	0.123*
Anticoagulant use	11 (5.4%)	9 (7.4%)	0.477*
Induction of labor	34 (17%)	26 (21%)	0.305*
Fail to labor progress	14 (6.9%)	10 (8.2%)	0.664*
Diabetes	31 (15%)	20 (16%)	0.788*
Preeclampsia	18 (8.9%)	14 (11%)	0.445*
Hypothyroidism	43 (21%)	26 (21%)	0.978*
Cesarean section	134 (66%)	78 (64%)	0.704*
Gestational age (weeks)	39 (38; 40)	39 (38; 40)	0.456***
BMI (kg/m2)	28.63 (26.08; 31.18)	27.74 (26.31; 31.09)	0.468***
Hematocrit count before birth (%)	37.80 (35.70; 39.75)	37.60 (35.92; 40.08)	0.932***
Duration of hospitalization (days)	6 (5; 6.5)	6 (5; 6.75)	0.935***
Blood units transfused (N)	1 (1; 1)	1 (1; 1)	0.009***
Peritoneal drainage	47 (23%)	0 (0%)	<0.001**
Postpartum hemorrhage	11 (5.4%)	1 (0.8%)	0.035**
Estimated blood loss (ml)	350 (300; 400)	350 (300; 400)	0.021***
Platelet count (10^3/µl)	196 (156; 236.5)	210 (181; 241.25)	0.011***
Hemoglobin concentration before birth (g/dl)	13 (12.15; 13.6)	12.4 (11.9; 13.3)	0.003***
Hemoglobin concentration after birth (g/dl)	11.6 (10.75; 12.5)	11.15 (10.50, 11.90)	0.005***
Decrease in hemoglobin concentration (g/dl)	1.2 (1.8; 0.6)	1.25 (2; 0.52)	0.865***
Hematocrit concentration after birth (%)	34.4 (32.1; 36.85)	33.75 (31.7; 36.68)	0.341***
Hematocrit concentration loss (%)	3 (5; 1.3)	3.5 (5.62; 2)	0.037***
Leukocyte count (10^3/µl)	9.20 (7.44; 10.85)	10.53 (9.29; 12.93)	<0.001***
Lymphocyte count (10^3/µl)	1.59 (1.20; 1.93)	1.87 (1.49; 2.2)	<0.001***
Neutrophile count (10^3/µl)	6.89 (5.24; 8.4)	7.61 (6.49; 9.68)	<0.001***
Sodium serum level (mmol/L)	139.00 (137; 140)	138.07 (137; 140)	0.993***
Potassium serum level (mmol/L)	4.09 (3.88; 4.28)	4.15 (3.98; 4.28)	0.122***
APTT (seconds)	29.90 (27.4; 32.4)	28 (26.50; 29.85)	<0.001***
INR	0.95 (0.92; 0.98)	0.97 (0.94; 1.02)	<0.001***
Birth weight (g)	3,450 (3,090; 3,770)	3,375 (3,097.5; 3,730)	0.916***

*Pearson's Chi-squared test; **Fisher's exact test; ***Mann-Whitney U test. Categorical variables are presented as N (%) and continuous variables are presented as Median (IQR).

Table S2. Univariate and multivariate logistic regression before propensity score matching

Variable	Univariate logistic regression			Multivariate logistic regression		
	OR	95% CI	p; value	OR	95% CI	p; value
COVID-19	4.19	1.44; 15.1	0.014	5.92	1.82; 23.9	0.006
≥ 3 labours	1.55	0.35; 4.95	0.500	0.71	0.14; 2.75	0.600
Previous operation on uterus	2.38	0.87; 6.77	0.090	2.67	0.8; 9.39	0.110
Anticoagulant use	3.55	0.78; 11.7	0.057	2.04	0.35; 8.89	0.400
Induction of labor	0.9	0.2; 2.94	0.900	2.23	0.43; 8.92	0.300
Fail to labour progess	0	-	> 0.900	0	-	> 0.900
Diabetes	1.38	0.31; 4.39	0.600	1.51	0.31; 5.63	0.600
Preeclampsia	0.67	0.04; 3.42	0.700	0.49	0.03; 2.93	0.500
Hypothyroidism	0.23	0.001; 1.16	0.200	0.13	0; 0.91	0.110
Cesarean section	3.94	1.09; 25.3	0.072	3.11	0.71; 22.4	0.20
Week of pregnancy	0.89	0.81; 0.98	0.013	0.88	0.8; 0.99	0.015
BMI	0.99	0.87; 1.1	0.800	0.99	0.87; 1.11	0.800
Hematocrit	0.99	0.85; 1.16	0.900	1	0.85; 1.18	>0.900
